# Fluoride Removal from Aqueous Solutions Using Poly(Styrene Sulfonate)/Nanoalumina Multilayer Thin Films

**DOI:** 10.1002/gch2.201700064

**Published:** 2018-01-16

**Authors:** Thanjavur Chandrasekaran Prathna, Ashok M. Raichur

**Affiliations:** ^1^ Department of Materials Engineering Indian Institute of Science Bangalore 560012 India; ^2^ Nanotechnology and Water Sustainability Research Unit University of South Africa The Science Campus Florida Park 1710 Roodepoort Johannesburg South Africa

**Keywords:** alumina, fluoride, LbL, polyelectrolyte, water

## Abstract

In the present study, fluoride removal from drinking water is investigated using layer‐by‐layer (LbL) fabricated poly(sodium 4‐styrene‐sulfonate) (PSS)/Al_2_O_3_ thin films. The surface morphology of the fabricated thin films is characterized using atomic force microscopy and field emission‐scanning electron microscopy. Optical profilometry is used to determine the self‐assembly of the multilayer thin films. The effect of various parameters such as adsorbent dosage, contact time, initial fluoride content, number of bilayers, surface area, and pH is thoroughly studied. Fluoride removal increases with the number of bilayers and number of slides (total surface area). The amount of fluoride adsorbed increases from 11.32 to 26 mg L^−1^ when the number of substrates increases from 1 to 5. A 68% removal of fluoride is observed when 20 bilayers of PSS/Al_2_O_3_ thin films with three slides at an initial fluoride concentration of 5 mg L^−1^ are used, thereby bringing down the fluoride concentration level below the World Health Organization permissible limit. Slide reusability studies reveal that the fabricated thin films can be used for ten cycles without affecting the fluoride removal properties of the film. This study demonstrates the potential application of immobilized PSS/Al_2_O_3_ thin films as an effective adsorbent for drinking water purification.

## 1 Introduction

Access to safe drinking water is a vital indicator of a country's development and recent years have witnessed a significant increase in the concentration levels of fluoride in surface waters.^1^, ^2^, ^3^ Natural and anthropogenic activities have led to increased fluoride contamination, which is now recognized as a major problem worldwide.^4^ Prolonged consumption of water contaminated with fluoride is detrimental and can lead to skeletal and dental fluorosis.^5^ There have been reports of fluoride concentration levels reaching up to ≈20 mg L^−1^ in several parts of the world, much above the permissible limit as put forth by the World Health Organization.^6^, ^7^

Adsorption is the most widely used method for fluoride removal from drinking water in view of its cost effectiveness, high efficiency, and simplicity of design among other defluoridation methods such as electro dialysis, ion exchange, Donnan dialysis, chemical precipitation, and reverse osmosis.^6^ Though various materials like activated alumina, activated carbon, other metal oxides, and low‐cost adsorbents have been used for the defluoridation of water, there are very few which take the concentration of fluoride below 1.5 mg L^−1^ level.^8^

Among the metal oxides, alumina (Al_2_O_3_) has been widely studied as a promising candidate for fluoride removal due to its excellent physical and textural properties when compared with other metal oxides.^9^, ^10^ However, using alumina in powder form as a sorbent has its practical limitations including difficulty in separation of powder from the liquid and leaching of metal/metal oxide into the treated water thereby incurring high treatment costs making the process economically unviable.^11^

In view of these drawbacks, there has been an increased interest in the fabrication of thin films. Films have been fabricated using sol–gel, chemical vapor deposition, spin coating, sputtering, and spray pyrolysis, to name a few. However these methods have their own limitations such as high‐temperature requirement, post‐treatment, and difficulty in controlling thickness. Layer‐by‐layer (LbL) multilayer assembly has been observed to be a novel thin film fabrication technique to deposit various types of materials on different substrates at room temperature.^12^ The method is based on the electrostatic attraction between oppositely charged polyelectrolytes or inorganic charged species such as metal oxides. One of the major advantages of this technique is its applicability to any kind of shape and substrate thereby making it an attractive option for scale up.^13^

Recently, polyelectrolytes have been extensively studied due to their wide applications such as multifunctional hybrid carrier systems, sensors, and in antireflective coatings, to name a few.^14^, ^15^, ^16^ Till date, there are a few reports on the application of LbL technique for the fabrication of TiO_2_/polyelectrolyte thin films for the degradation of organic pollutants/dyes and also on the application of silver nanoparticle/polyelectrolyte thin films for the removal of coliform bacteria.^13^, ^17^, ^18^, ^19^, ^20^, ^21^ Recent studies have also reported the fabrication of LbL‐assembled diamond‐based core–shell nanocomposites for dye degradation,^22^ but there are hardly any studies on the application of LbL technique for defluoridation of drinking water.

Though there are numerous reports on the application of alumina for fluoride removal,^6^ nearly all studies were carried out at higher initial concentrations of fluoride since the lower limit of fluoride reduction is greater than 2 mg L^−1^.^8^

Thus the primary objective of this work was to demonstrate the utility of LbL technique for the fabrication of polyelectrolyte (PSS)/Al_2_O_3_ thin films and to determine its application for the defluoridation of drinking water. The main advantage of using PSS/Al_2_O_3_ thin films was due to their highly ordered nonporous structure and relative hardness.^23^ The LbL‐fabricated film was also compared with films prepared by other methods such as sol–gel to show its effectiveness in terms of surface morphology and defluoridation capacity. Fluoride removal studies were carried out at lower initial concentration levels of fluoride (1–5 mg L^−1^) to demonstrate the effectiveness of the method in removing fluoride below the permissible limit. The method was thus intended to be put to use as a secondary or tertiary treatment system for drinking water purification.

## 2 Results and Discussion

### 2.1 Characterization of As‐Received α Al_2_O_3_ Nanoparticles

The scanning electron micrograph of Al_2_O_3_ nanoparticles suggested the presence of nearly spherical particles of size around 100 nm. The crystal structure of as‐received particles analyzed using X‐ray diffraction (XRD) confirmed the presence of predominant α phase. The average crystallite size was calculated (by Debye–Scherrer equation) to be 38 ± 3 nm and was close to the crystallite size mentioned by the supplier. The particle size distribution of the as‐received nanoparticles was determined to be around 164 ± 1.20 nm. The point of zero charge of α Al_2_O_3_ was determined by zeta potential studies and it was observed to be around pH 7.4. Therefore pH below the isoelectric point of α Al_2_O_3_ was taken for the fluoride removal studies to ensure that the surface of the metal oxide nanoparticle carried a positive charge.

### 2.2 Fabrication of Thin Films

#### 2.2.1 Characterization of Thin Films

**Figure 1:**
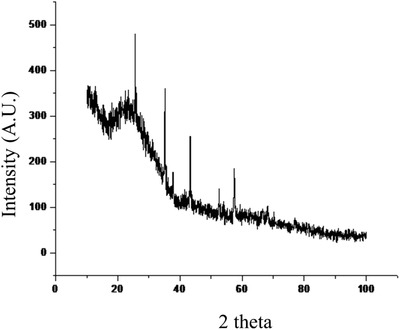
XRD spectra of LbL fabricated PEI(PSS/Al_2_O_3_) thin films.

Thin films were fabricated using the LbL technique in which polyethylenimine (PEI) was added as the precursor monolayer to reverse the charge of the substrate. Application of branched PEI as a monolayer is also known to enhance the thickness, stability, and uniformity of polyelectrolyte multilayers.^24^ The fabricated thin films were characterized using XRD. **Figure** **1** shows the XRD pattern of PSS‐Al_2_O_3_ films fabricated by LbL technique. The thin films exhibited strong XRD peaks at 2θ values close to 35°, 45°, and 58° confirming the presence of α phase of alumina.^25^

**Figure 2:**
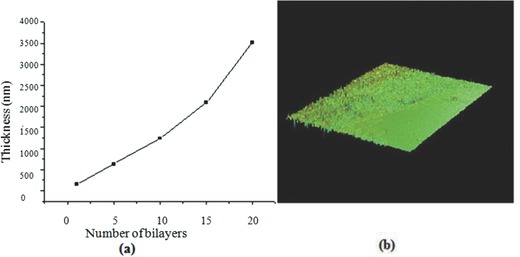
Optical profilometry studies showing a) the number of bilayers versus film thickness and b) 3D profile view of PEI(PSS/Al_2_O_3_)_10_.

The thickness of the multilayer thin films was studied using a noncontact optical profiling system. **Figure** **2**a shows the change in film thickness with increase in the number of PSS‐Al_2_O_3_ bilayers. The total thickness of PSS‐Al_2_O_3_ film having 5 and 15 bilayers was 632 and 2091 nm, respectively. It could be observed that the film thickness increased almost linearly with increase in the number of bilayers from 1 to 20 showing uniform growth of the polyelectrolyte/Al_2_O_3_ thin films. The increase in thickness may also depend on the nature of the polyelectrolytes used, the pH of deposition, and the average size of the particles.^13^ Figure 2b shows the 3D profile view of a ten‐bilayer PSS‐Al_2_O_3_ film with thickness of 1239 nm. The film depicts uniform growth of the multilayer films with a clear interface between the coated and uncoated surfaces.

**Figure 3:**
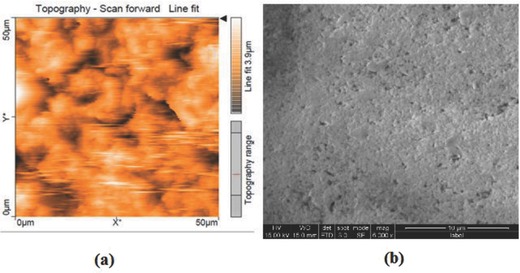
a) AFM image and b) FE‐SEM image (top view) of PEI(PSS/Al_2_O_3_)_20_ fabricated through LbL route.

The PSS‐Al_2_O_3_ films were further characterized by atomic force microscopy (AFM) and field emission‐scanning electron microscopy (FE‐SEM) to evaluate the surface morphology of the multilayer films. **Figure** **3**a shows the AFM micrograph of the surface of 20‐bilayer PSS‐Al_2_O_3_ film. It was observed that the roughness of the film was 3.9 µm. Figure 3b shows the top view FE‐SEM image of LbL‐coated multilayer films. The micrograph showed a smooth surface morphology and a well‐formed network of polyelectrolytes and nanoparticles evenly distributed throughout.

#### 2.2.2 Comparison of LbL‐Al_2_O_3_ Films with Other Films

Figure S1a,b shows the top view FE‐SEM micrographs of as‐received Al_2_O_3_ nanoparticles coated in the absence of polyelectrolytes and nanoparticles synthesized by the sol–gel process, respectively. The cross section of the thin films prepared by the above‐mentioned methods is shown in Figure S2a,b, respectively. It was evident from the micrographs that these methods did not lead to a uniform coating and resulted in the formation of clusters of polyelectrolytes and Al_2_O_3_ in patches.

### 2.3 Fluoride Removal Studies

**Figure 4:**
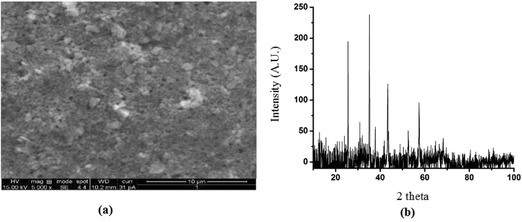
a) FE‐SEM image and b) XRD spectra of fluoride sorbed PEI(PSS/Al_2_O_3_) thin film.

**Figure** **4**a,b shows the FE‐SEM and XRD images of fluoride sorbed LbL‐Al_2_O_3_ films. No significant changes could be observed on the films upon fluoride adsorption. The various factors affecting fluoride adsorption are discussed in the following sections.

#### 2.3.1 Effect of Contact Time

**Figure 5:**
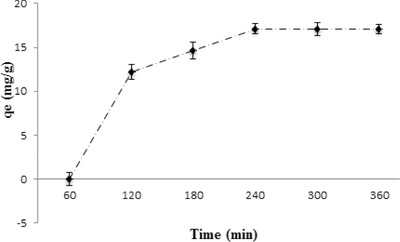
Effect of contact time on fluoride sorption on LbL‐Al_2_O_3_ films.

The sorption of fluoride on LbL‐Al_2_O_3_ films was investigated as a function of contact time (**Figure** **5**) at a fixed initial fluoride concentration (5 mg L^−1^). As the contact time increased from 1 to 2 h, the sorption capacity increased steadily up to 12.2 mg L^−1^ after which there was a gradual increase in the amount of fluoride sorbed. The equilibrium time was observed to be at the end of 4 h when around 17.1 mg g^−1^ of fluoride was adsorbed onto the thin film. This study indicated that maximum amount of fluoride ions was adsorbed within the first 120 min, which might be due to excess availability of vacant sites. The almost saturated sorption capacity beyond 240 min indicates that there were no more active sites left for sorption. For further studies, equilibration time was fixed at 4 h.

#### 2.3.2 Effect of Adsorbent Dose

**Figure 6:**
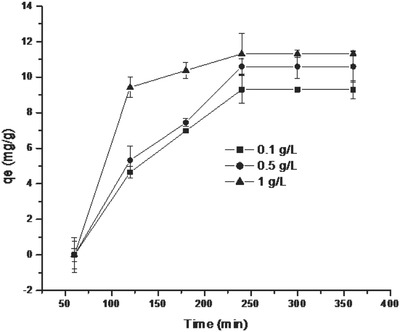
Effect of adsorbent dose on fluoride sorption on LbL‐Al_2_O_3_ films.

Increase in adsorbent dose from 0.1 to 1 g L^−1^ led to an increase in adsorption capacity (**Figure** **6**). The amount of fluoride sorbed was observed to increase from 9.3 to 11.32 mg g^−1^ when the adsorbent dose increased from 0.1 to 1 g L^−1^. This increase in defluoridation capacity was due to the increased availability of the reaction sites in the adsorbent. For further studies, adsorbent dose of 1 g L^−1^ was used.^26^, ^27^

#### 2.3.3 Effect of pH

**Figure 7:**
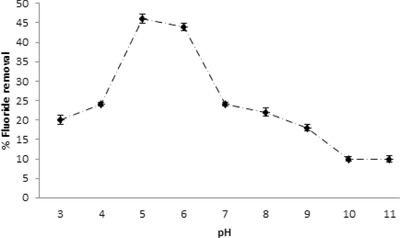
Effect of pH on fluoride sorption on LbL‐Al_2_O_3_ films.

pH is a major factor that controls adsorption at the water–adsorbent interface and the charge density, which varies with pH, is one of the major parameters in determining fluoride load onto the surface of the adsorbent.^28^ Batch adsorption experiments were conducted at pH ranging from 3 to 11 at fixed initial fluoride concentration (5 mg L^−1^) and the sorption capacities of LbL‐Al_2_O_3_ thin films were measured (**Figure** **7**). The maximum defluoridation capacity was observed at acidic pH ranges and the minimum at alkaline pH. Initially, the defluoridation capacity increased with increase in pH and reached a maximum at pH 5 (46%) after which it decreased steadily (10% at pH 11). At pH < pH_pzc_ (7.4), the surface of Al_2_O_3_ is positively charged and thereby attracts negatively charged fluoride ions. The significant decrease in defluoridation at alkaline pH was probably due to competition from negatively charged hydroxyl ions, which in general leads to a decrease in surface density of AlOH_2_^+^ sites and increase in AlO^−^ sites.^29^, ^30^ Minimal defluoridation was also observed at low pH (pH 3) as highly acidic conditions promoted the formation of hydrofluoric acid (HF) thereby leading to dissolution of Al_2_O_3_.^31^ It has also been previously reported that α‐Al_2_O_3_ on its own had increased solubility below pH 5 studied through the construction of a pH solubility diagram.^32^

#### 2.3.4 Effect of Surface Area

**Figure 8:**
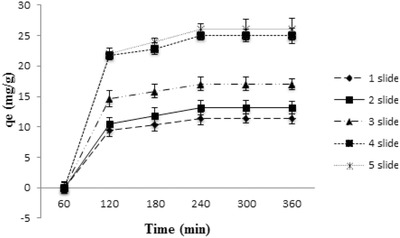
Effect of surface area on fluoride sorption on LbL‐Al_2_O_3_ films.

To study the effect of surface area on fluoride removal, experiments were carried out with 1, 2, 3, 4, 5 immobilized thin films with a single bilayer of PEI (PSS/alumina) at pH 5, contact time = 4 h, adsorbent dose = 1 g L^−1^, and the initial concentration of fluoride was 5 mg L^−1^ (**Figure** **8**). It was observed that the fluoride removal efficiency enhanced when more than one substrate was used. The amount of fluoride sorbed (*q*_e_) increased from 11.32 mg g^−1^ when one slide was used to 17.07 mg g^−1^ when three slides were used. However the increase in defluoridation capacity was not linear with the increase in the number of slides. This could be attributed to the fact that all slides might not be oriented in an identical manner for enhanced interaction with fluoride.

#### 2.3.5 Effect of Bilayers

**Figure 9:**
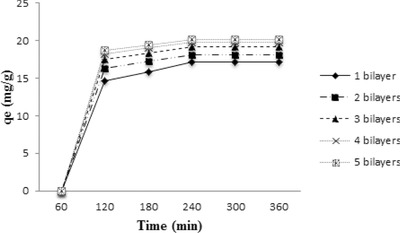
Effect of the number of bilayers on fluoride sorption on LbL‐Al_2_O_3_ films.

**Figure** **9** shows the effect of the number of bilayers on fluoride removal. This was done by varying the number of bilayers from 1 to 20 (concentration of Al_2_O_3_ = 1 g L^−1^, the number of immobilized catalysts = 3, contact time = 4 h, and concentration of initial fluoride = 5 mg L^−1^). The amount of fluoride sorbed from the solution (*q*_e_) increased from 17.1 mg g^−1^ for one bilayer to 20.15 mg g^−1^ for 20 bilayers. It could be observed that the fluoride removal increased with the number of bilayers. This could be attributed to the fact that as the number of bilayers increased, there was an increase in the concentration of immobilized adsorbent, hence increased fluoride removal. This study also established that fluoride removal was carried out not only by the outermost Al_2_O_3_ layer but also by the inner layers of alumina. It was also significant to observe that the increase in defluoridation capacity was not linear with the increase in the number of bilayers implying that the efficacy of Al_2_O_3_ in the innermost layers was not the same as the outer surface. This result was consistent with previous studies by Priya et al.^17^ in which the authors observed that increase in the number of bilayers of titania led to an increase in photocatalytic degradation of dimethoate, though not linear.

**Figure 10:**
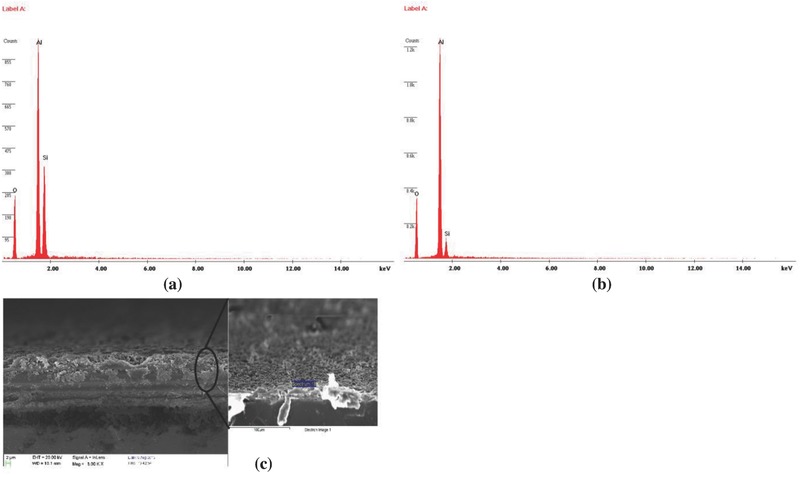
EDAX spectra of PEI(PSS/Al_2_O_3_) thin film with a) five and b) ten bilayers and c) FE‐SEM micrograph of PEI(PSS/Al_2_O_3_)_5_ thin film.

**Figure** **10**a,b shows the energy dispersive analysis X‐ray spectrometer (EDAX) spectra of LbL Al_2_O_3_ thin films with five and ten bilayers, respectively. EDAX spectra of fabricated thin films with five bilayers had 43.71 wt % Al while those films with ten bilayers had 62.36 wt% of Al as seen from the images. Figure 10c represents the cross sectional FE‐SEM image showing five bilayers. The image further confirmed the presence of Al in layers.

**Figure 11:**
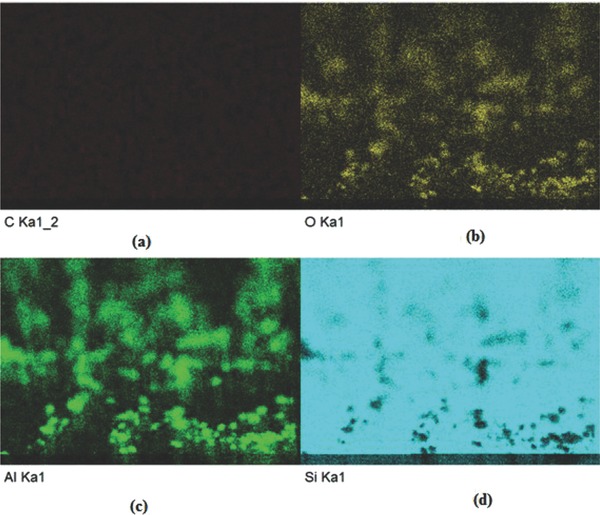
a–d) EDAX mapping of PEI(PSS/Al_2_O_3_)_5_ thin film.

**Figure** **11**a–d shows the EDAX mapping (done by secondary electron scattering) of fabricated thin films with five bilayers. The images showed the presence of C, Al, and O in the samples. The presence of C was due to polyelectrolytes in the fabricated thin films.

#### 2.3.6 Effect of Initial Fluoride Concentration

**Figure 12:**
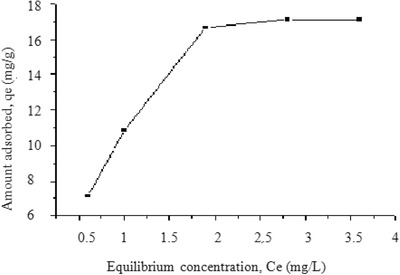
Fluoride sorption on PEI(PSS/Al_2_O_3_) thin film.

In order to determine the sorption capacity of LbL‐Al_2_O_3_ film for fluoride, equilibrium sorption of fluoride was studied as a function of fluoride concentration (**Figure** **12**). With increase in initial fluoride concentration from 1 to 5 mg L^−1^, both fluoride equilibrium concentration and equilibrium adsorption capacity exhibited increasing trends. A high concentration has been observed to impart a driving force to overcome the mass transfer resistance between aqueous and solid phase.^33^ Figure 12 shows that the sorption capacity at equilibrium gradually increased with increase in equilibrium fluoride concentration denoting availability of readily accessible sites for sorption initially. Site saturation occurs much later with further increase in fluoride concentration leading to the formation of a plateau. Similar results were observed by Kumar et al. and the sorption capacity in their case was observed to be 14 mg g^−1^ for fluoride on nanoalumina at 25 °C.^6^

#### 2.3.7 Effect of Other Anions

Since contaminated groundwater usually contains other co‐existing anions that might compete with fluoride for active sites, it was imperative to study the effect of competing anions on fluoride removal. Hence we determined the effect of chloride, nitrate, sulphate, carbonate, and phosphate anions on fluoride removal by LbL‐fabricated Al_2_O_3_ thin films at 5 mg L^−1^ initial fluoride concentration. The concentration of competing anions was 5 and 10 mg L^−1^.

**Figure 13:**
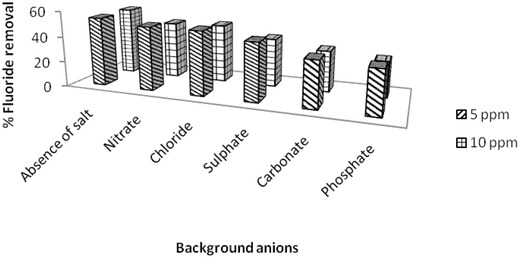
Effect of competing anions on fluoride sorption.

**Figure** **13** shows the effect of various anions on fluoride sorption on Al_2_O_3_ thin films. Increase in the concentration of anions (from 5 to 10 mg L^−1^) led to a corresponding decrease in % fluoride removal. Fluoride sorption was largely influenced by the presence of phosphate, sulphate, and carbonate while the least interfering ions were nitrate and chloride. Similar results were observed by Kumar et al. on their studies on fluoride removal using nanoalumina in which they observed that inner spherically sorbing anions (phosphate and sulphate) significantly competed for the limited amount of sorption sites on alumina while outer spherically absorbing anions (nitrate and chloride) had least interference on fluoride removal.^6^ The % fluoride removal decreased from 54% to 28% in the presence of 10 mg L^−1^ phosphate. However, the competition of phosphate with fluoride for active sites might not be a major concern since its content in groundwater has been reported to be usually less than 10 mg L^−1^.^34^ Presence of carbonate in the fluoride solution led to increase in pH of the solution thus lowering the number of active sites for fluoride sorption.^35^

#### 2.3.8 Adsorbent Loading

The amount of adsorbent deposited on each slide was determined by calculating the difference between the empty and the weight after each layer of deposition (excluding the weight of PSS). On average, the amount of Al_2_O_3_ deposited per each layer was ≈ 0.22 mg and the average deposition per slide after ten bilayers was ≈2.36 mg. Since three slides were used for the study, a total of 7.08 mg of Al_2_O_3_ was required to sorb 19.17 mg of fluoride per gram of Al_2_O_3_. When the same amount of adsorbent (7.08 mg) was used in suspension form, the fluoride sorbed was calculated to be 10.83 mg g^−1^.

### 2.4 Fluoride Sorption Mechanism

The mechanism of fluoride sorption has been extensively studied and has been observed to be governed by electrostatic adsorption and complexation. The positive charge at the surface of alumina attracts negatively charged fluoride by means of electrostatic attraction. The positive surface might also attract fluoride by complexation since fluoride acts as a chelating agent.^35^, ^36^

### 2.5 Slide Reusability

**Figure 14:**
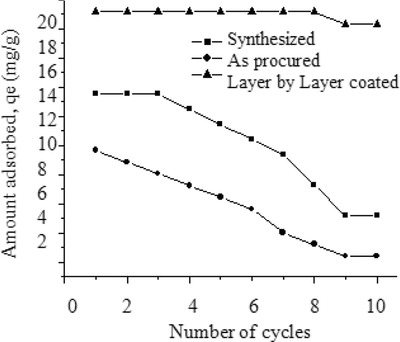
The number of cycles versus fluoride sorbed on fabricated thin films prepared by various methods.

Slide reusability studies were performed on Al_2_O_3_ thin films immobilized by LbL technique. The results were compared to thin films prepared using (1) procured Al_2_O_3_ (in the absence of polyelectrolytes) and (2) Al_2_O_3_ synthesized and coated using sol–gel method. The experiments were conducted under the following conditions: Al_2_O_3_ = 1 g L^−1^, fluoride concentration = 5 mg L^−1^, number of slides = 3, number of bilayers = 10, and contact time = 4 h. The defluoridation capacity of the immobilized substrate after ten cycles was determined in the current study. The results are shown in **Figure** **14**. It could be observed that there was a notable decrease in the amount of fluoride sorbed when thin films prepared using as‐received Al_2_O_3_ nanoparticles and synthesized nanoparticles coated by the sol–gel process were used. The amount of fluoride sorbed (*q*_e_) decreased from 9.68 to 2.42 mg g^−1^ for as‐received Al_2_O_3_ and from 13.54 to 5.21 mg g^−1^ when synthesized Al_2_O_3_ was used. There was no change in the amount of fluoride sorbed until eight cycles (19.17 mg g^−1^) when LbL Al_2_O_3_ thin films were used after which there was a marginal decrease in defluoridation capacity (18.33 mg g^−1^).

**Figure 15:**
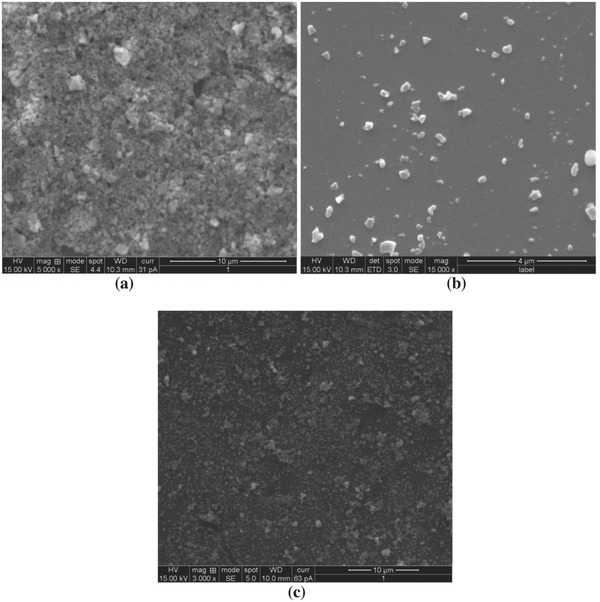
FE‐SEM image of thin film prepared by a) LbL, b) as‐received particles, and c) sol–gel after ten cycles.

**Figure** **15**a–c shows the FE‐SEM micrograph of LbL‐coated, as‐received, and synthesized Al_2_O_3_ films after ten cycles, respectively, while Figure 15a–c shows the corresponding EDAX spectra of the same. LbL‐coated Al_2_O_3_ with ten bilayers had a 2.49% loss of Al whereas the as‐received Al_2_O_3_ nanoparticles coated in the absence of polyelectrolytes had a 90.51% loss of Al after ten cycles. No significant changes in the surface characteristics of LbL Al_2_O_3_ thin films were observed from the FE‐SEM images while there was a significant loss of Al_2_O_3_ for thin films prepared using the other two processes thus indicating the stability of LbL‐coated films after several cycles. These studies are significant in terms of cost effectiveness since it drastically reduces the cost of input materials.

Though there are numerous studies on the removal of fluoride using adsorbents in powder form, there are hardly few studies on the reusability of the adsorbent. In our study we also observed that defluoridation capacity of thin films was greater than that of the same adsorbent when used in powder form. The films were observed to possess the same capacity even after ten cycles of usage. A major drawback of using adsorbent in powder form is the separation of adsorbent after the process of fluoride removal. A centrifugation step is thus required to remove the adsorbent from the system thus proving to be a deterrent during scale up. In the case of LbL Al_2_O_3_ thin films, the treated solutions were clear of suspended nanoparticles as seen from EDAX analysis. The study can be further extended to continuous systems as part of scale up operations.

## 3 Conclusion

A novel LbL Al_2_O_3_ thin film was fabricated, characterized, and applied in fluoride removal from drinking water. The fabricated thin film was compared to other methods such as sol–gel to demonstrate its efficiency in effective fluoride removal. A 68% removal of fluoride was observed when 20 bilayers of PSS/Al_2_O_3_ thin films with three substrates at an initial fluoride concentration of 5 mg L^−1^ was used thereby bringing down the fluoride concentration level below the World Health Organization permissible limit. Increase in the number of bilayers could substantially lead to better defluoridation capacity and decreased contact time. Further studies would be directed toward scale up of the process and on different substrates. The current study is an answer with regard to the drawbacks of using nanomaterials in powder form for water purification. The method has potential advantages such as substrate reusability, treated solution being free of the adsorbent, etc., which is very important in terms of overall cost of treating water. The study thus demonstrates potential utility of the fabricated nano‐Al_2_O_3_ thin film as a promising substrate for secondary and tertiary water purification system.

## 4 Experimental Section

*Materials*: Poly(sodium 4‐styrene‐sulfonate) (PSS, *M*_W_ = 70 000 g mol^−1^), PEI branched (*M*_W_ = 25 000 g mol^−1^), sodium fluoride, and aluminum tri‐sec‐butoxide were obtained from Sigma‐Aldrich (USA). Nanocrystalline α alumina powder was obtained from Inframat Advanced Materials (USA). The physicochemical parameters as reported by the supplier were: crystal size ≈40 nm and particle size ≈150 nm. Dispersions of Al_2_O_3_ nanoparticles of different concentrations were freshly prepared by ultrasonic vibrations for 10 min for coating studies. All other chemicals used were of analytical grade. Microscopic glass slides (75 × 25 mm) were used as substrates for coating.

*Characterization of As‐Received Particles*: The morphology of the nanoparticles was observed using a scanning electron microscope while the average crystallite size was determined by X‐ray diffraction analysis. The details of the instrument are provided in section “*Fabrication of PSS/Al_2_O_3_ Thin Films*.” The size distribution and zeta potential of the nanoparticles was determined using a Zetasizer (Zetasizer Nanoseries ZEN‐3690, Malvern Instruments, UK).

*Fabrication of PSS/Al_2_O_3_ Thin Films*: In the current study, multilayer PSS/Al_2_O_3_ thin films were immobilized using LbL technique described by Decher and Hong.^12^ Accordingly, microscopic glass slides that served as substrates for coating were cleaned by sonicating in a solution containing 2:1 (v/v) ratio of isopropanol and water. PSS and PEI (of concentration 1 g L^−1^ in deionized water) were prepared and adjusted to desired pH (pH 3). The pH of water used as rinse solution was adjusted to that of polyelectrolytes (pH 3). To deposit Al_2_O_3_ on glass substrates, a dispersion of 1 g L^−1^ of the oxide nanoparticle in deionized water was prepared with its pH adjusted (pH 3) so as to ensure that it had the desired charge to be suitable for LbL technique.

Initially, one precursor layer of PEI was deposited for 15 min to reverse the charge on the substrate followed by rinsing with water for 3 min. Alternate multilayers of PSS/Al_2_O_3_ were then assembled by immersing the glass substrates in PSS and Al_2_O_3_ alternately. The topmost layer was Al_2_O_3_ in all cases. The multilayer thin films were coated using a dip coater (KSV Dipcoater LM, Finland).

The desired pH for coating was arrived at taking into account the isoelectric point of the nanoparticle. The isoelectric point of α alumina was experimentally determined by zeta potential studies to be around 7. Al_2_O_3_ particles are stable and positively charged below the isoelectric point. PSS is a strong polyelectrolyte that is negatively charged at all pH while PEI is a weak cationic polyelectrolyte and strongly ionized at low pH (<pH 6).

To demonstrate the effectiveness of LbL technique in terms of catalyst immobilization, it was compared with other methods, in which (1) α Al_2_O_3_ nanoparticle dispersion (1 g L^−1^) was directly coated onto glass slides in the absence of polyelectrolytes and (2) Al_2_O_3_ films were synthesized and deposited by sol–gel technique using aluminum tri‐sec‐butoxide as precursor.^37^

*Fabrication of PSS/Al_2_O_3_ Thin Films—Characterization of Thin Films*: The fabricated thin films were characterized by the following techniques:

X‐ray diffraction: The thin films were subjected to X‐ray diffraction analysis using an X‐ray diffractometer (PANalytical‐XPERT PRO diffractometer system, Eindhoven, the Netherlands) with the target being Cu K_α_ with a wavelength of 1.54060 Å. The generator was operated at 40 kV with a 30 mA current. The scanning range was selected between 10^о^ and 100^о^.

Atomic force microscopy: The surface of the multilayer thin films was examined using atomic force microscope (Nanosurf Easyscan 2, Switzerland) in noncontact mode.

Field emission‐scanning electron microscopy‐energy dispersive analysis X‐ray spectrometer: The surface morphology and thickness of the thin film was studied using FE‐SEM (Supra 55 Zeiss, USA). The elemental composition of the LbL‐coated multilayer thin films was determined using EDAX. The thin films were coated on silica substrate and sputtered with Au before analysis.

Optical profilometer: The thickness of the fabricated thin films was also measured using a noncontact optical profilometer (Wyko NT 1100, Optical Profiling System, Veeco Instruments, USA). The thin films were fabricated on a silica substrate for this study.

*Fluoride Sorption Studies*: The sorption of fluoride on fabricated thin films was conducted at room temperature by batch studies. To fluoride solution (50 mL) of varying initial concentration, fabricated thin alumina thin films were added and interacted for a specified period of contact time. Samples were withdrawn periodically and were centrifuged at 10 000 rpm for 10 min. The supernatant was then filtered using a 0.45 µ filter and the resultant solution was analyzed for residual fluoride concentration using a fluoride ion selective electrode (Orion, Thermo Scientific, USA). To avoid interference of other ions on electrode performance, a total ionic strength fixer and buffer solution (TISAB III) was added to a given volume of fluoride containing supernatant before actual measurements.

The amount of fluoride sorbed (*q*_e_ in mg g^−1^) was calculated as follows

(1) $$q_{e}=\frac{(C_{o}-C_{e})V}{m}$$

(2) $$\mathrm{Biosorption}\;\%=\frac{C_{o}-C_{e}}{C_{o}}\times \mathrm{100}$$

where *C*_o_ and *C*_e_ are the initial and final concentrations of fluoride in solution (mg L^−1^), *V* is the volume of solution (L), and *m* is mass of sorbent.

In order to determine the optimum process parameters for the system, the factorial design of experiments was employed in the current study. This design of experiment implied that one experimental factor was varied at a time keeping the others constant. Process parameters such as concentration of nanoparticle in suspension (0.1, 0.5, and 1 g L^−1^), the number of bilayers (1, 5, 10, 15, and 20), effect of surface area (number of slides 1–5), initial fluoride concentration (1–5 mg L^−1^), contact time (60–360 min), and initial pH of the system (3–11) were varied in the present study. The effect of competing anions (nitrate, chloride, sulphate, carbonate, and phosphate) on fluoride sorption was also studied under a fixed fluoride concentration (5 mg L^−1^) and initial anion concentrations of 5 and 10 mg L^−1^ in the presence of 1 g L^−1^ sorbent coated as thin film.

## Conflict of Interest

The authors declare no conflict of interest.

## Supporting information

Supplementary
