# Supplementary material for: Fluoride Removal from Aqueous Solutions Using Poly(Styrene Sulfonate)/Nanoalumina Multilayer Thin Films
Source: Glob Chall. 2018 Jan 16;2(2):1700064. doi: 10.1002/gch2.201700064 (PMC6607118; doi:10.1002/gch2.201700064)

Copyright WILEY-VCH Verlag GmbH & Co. KGaA, 69469 Weinheim, Germany, 2018.

Supporting Information

for *Global Challenges,* DOI: 10.1002/gch2.201700064

Fluoride Removal from Aqueous Solutions Using Poly(Styrene Sulfonate)/Nanoalumina Multilayer Thin Films

Thanjavur Chandrasekaran Prathna and Ashok M. Raichur*

Enhanced Defluoridation from aqueous solutions using Layer-by Layer (LbL) Fabricated Poly (Styrene Sulfonate)/Al_2O_3 Multilayer Thin Films

Prathna T.C.[a] and Ashok M. Raichur [a,b]

[a]Department of Materials Engineering, Indian Institute of Science, Bangalore-560012, India.

[b]Nanotechnology and Water Sustainability Research Unit, University of South Africa, Science Campus, Florida, Johannesburg, South Africa

Figure S1a: FE-SEM micrograph of as received alumina nanoparticles coated as thin film in the absence of polyelectrolytes (top view)

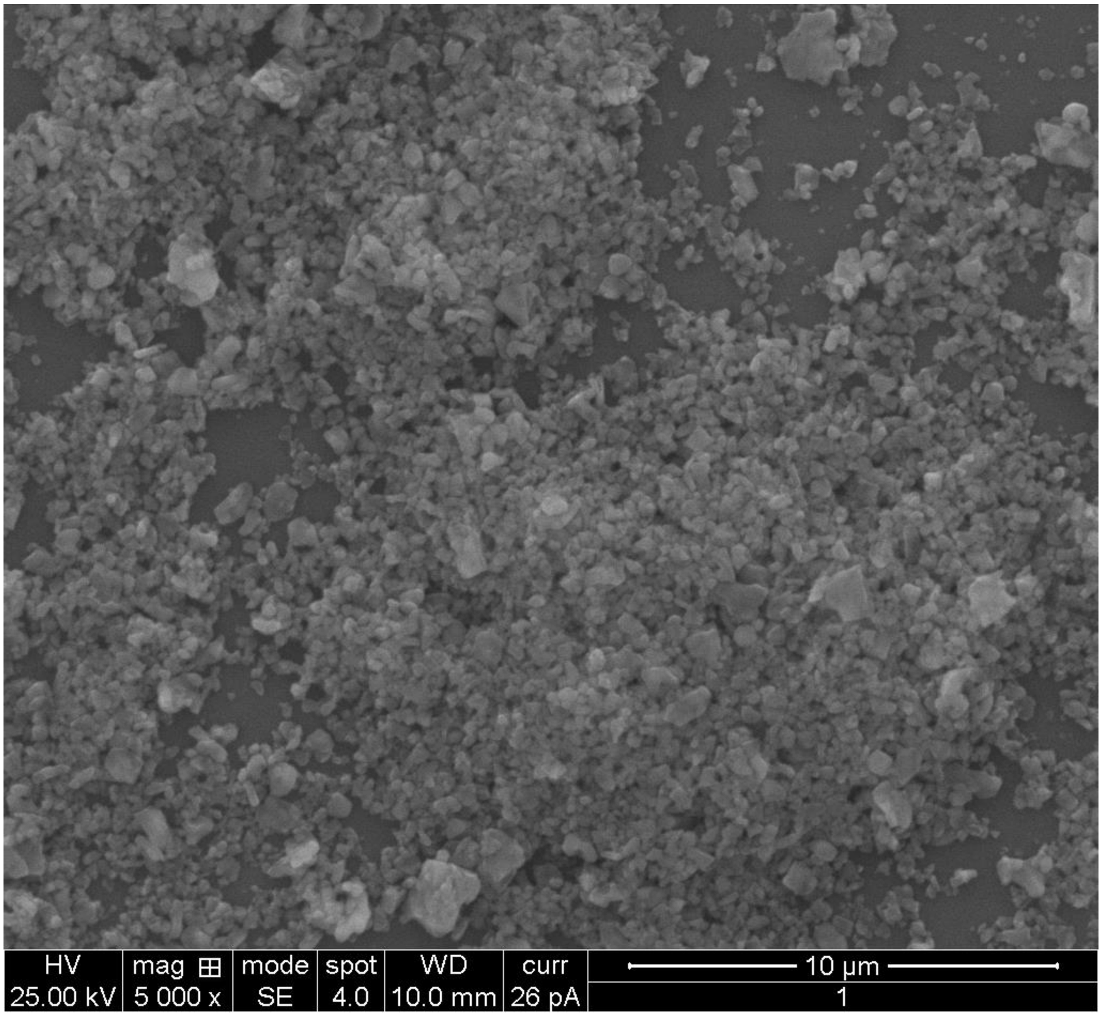

Figure S1b: FE-SEM micrograph of alumina nanoparticles synthesized and coated by sol-gel process (top view)

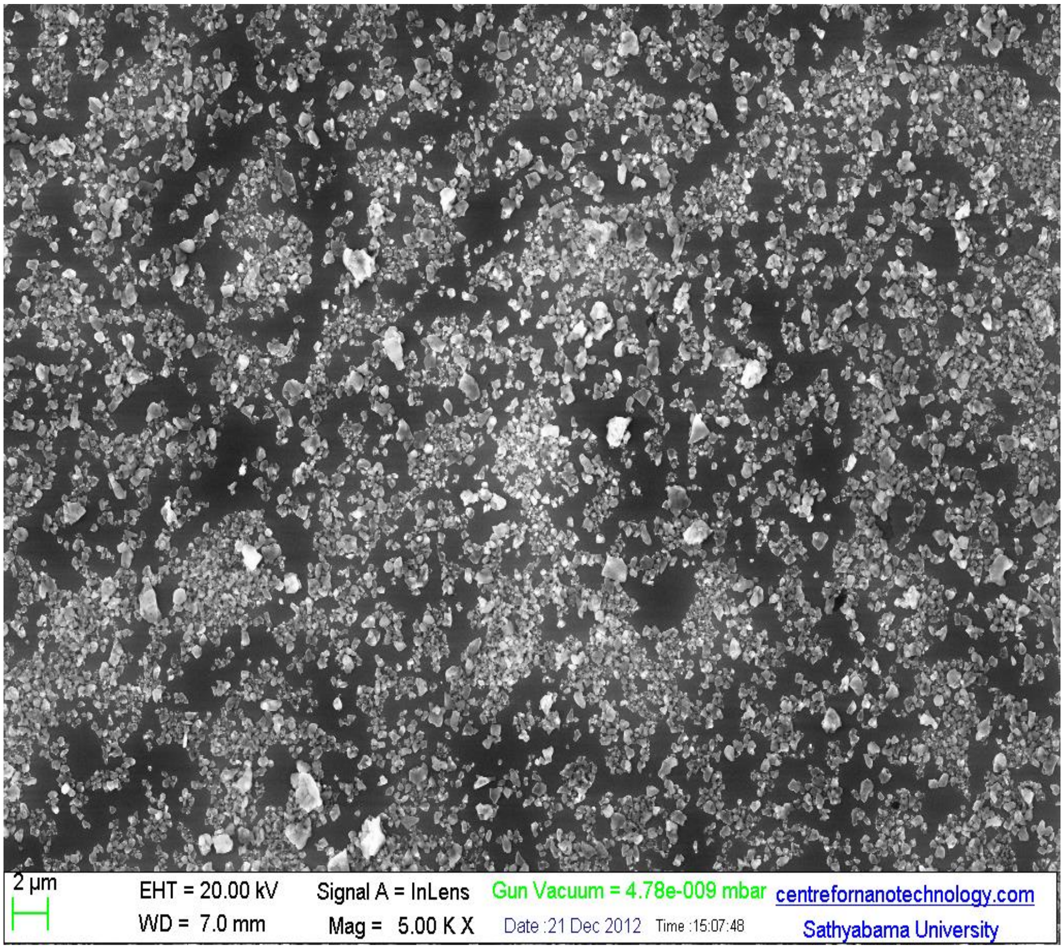

Figure S2a: FE-SEM micrograph of as received alumina nanoparticles coated as thin film in the absence of polyelectrolytes (cross section)

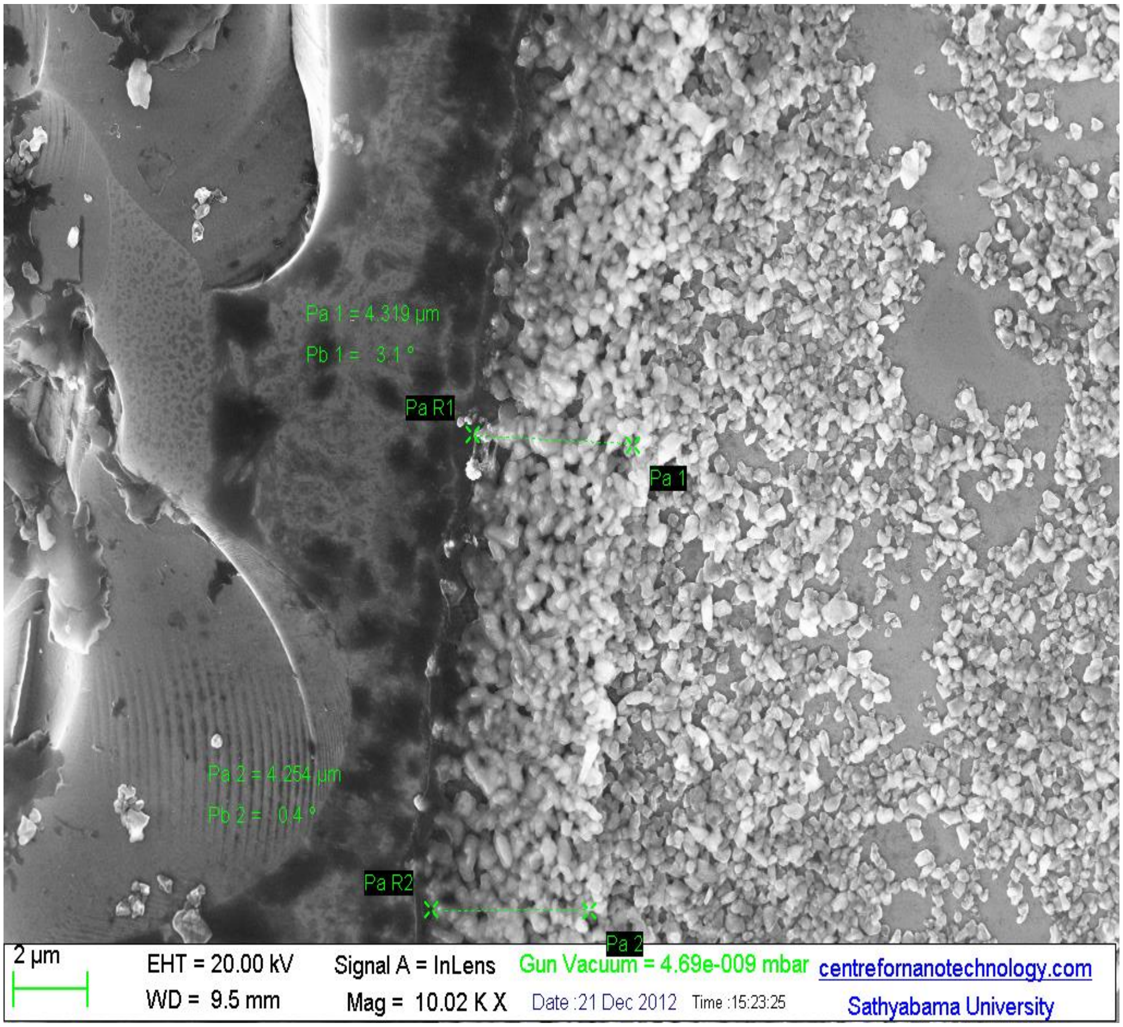

Figure S2b: FE-SEM micrograph of alumina nanoparticles synthesized and coated by sol-gel process (cross section)

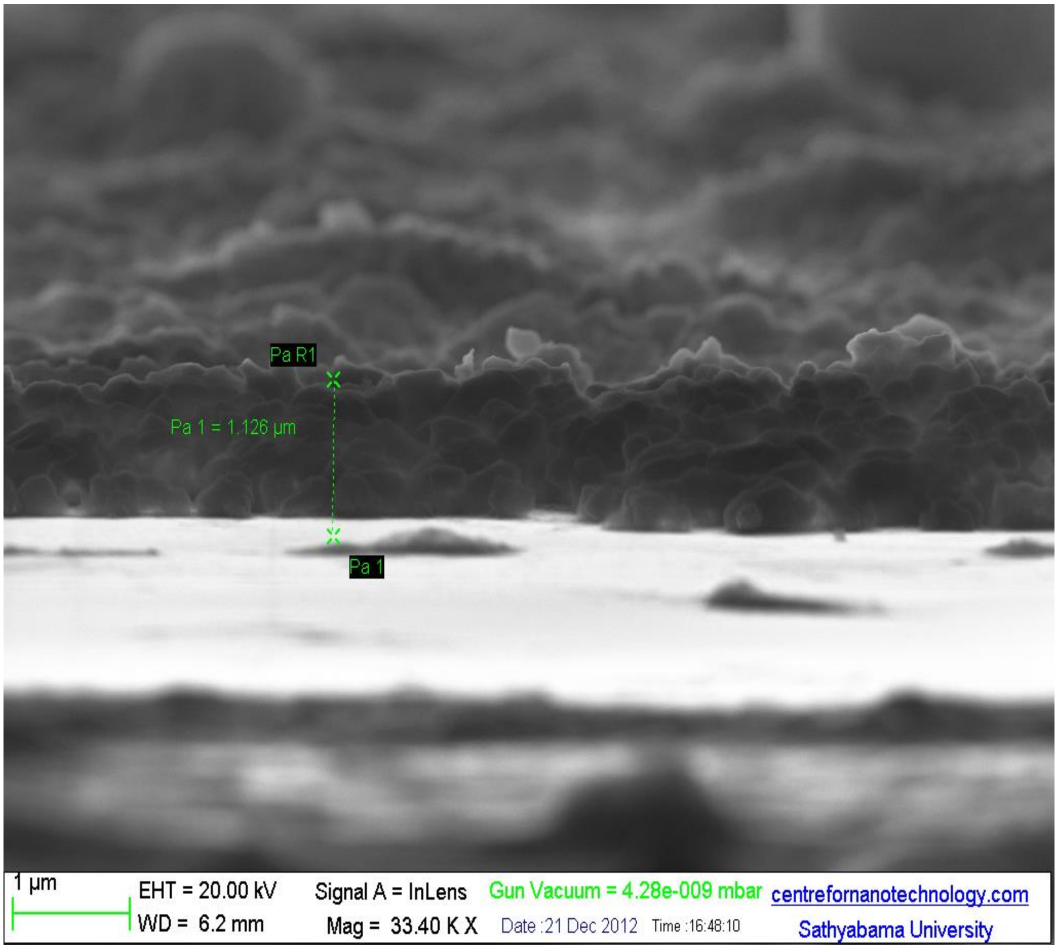

Supplement: Supplementary file 1 — Supplementary [file GCH2-2-1700064-s001.pdf]
